# Neural Correlates of Learning from Induced Insight: A Case for Reward-Based Episodic Encoding

**DOI:** 10.3389/fpsyg.2016.01693

**Published:** 2016-11-01

**Authors:** Jasmin M. Kizilirmak, Hannes Thuerich, Kristian Folta-Schoofs, Björn H. Schott, Alan Richardson-Klavehn

**Affiliations:** ^1^Cognitive Neuroscience Lab, Institute of Psychology, University of HildesheimHildesheim, Germany; ^2^Memory and Consciousness Research Group, Department of Neurology, Otto-von-Guericke University of MagdeburgMagdeburg, Germany; ^3^Leibniz Institute for Neurobiology, Department of Behavioral NeurologyMagdeburg, Germany; ^4^Department of Psychiatry, Charité University HospitalBerlin, Germany

**Keywords:** problem solving, long-term memory, encoding, fMRI, hippocampus, mPFC, insight

## Abstract

Experiencing insight when solving problems can improve memory formation for both the problem and its solution. The underlying neural processes involved in this kind of learning are, however, thus far insufficiently understood. Here, we conceptualized insight as the sudden understanding of a novel relationship between known stimuli that fits into existing knowledge and is accompanied by a positive emotional response. Hence, insight is thought to comprise associative novelty, schema congruency, and intrinsic reward, all of which are separately known to enhance memory performance. We examined the neural correlates of learning from induced insight with functional magnetic resonance imaging (fMRI) using our own version of the compound-remote-associates-task (CRAT) in which each item consists of three clue words and a solution word. (Pseudo-)Solution words were presented after a brief period of problem-solving attempts to induce either sudden comprehension (CRA items) or continued incomprehension (control items) at a specific time point. By comparing processing of the solution words of CRA with control items, we found induced insight to elicit activation of the rostral anterior cingulate cortex/medial prefrontal cortex (rACC/mPFC) and left hippocampus. This pattern of results lends support to the role of schema congruency (rACC/mPFC) and associative novelty (hippocampus) in the processing of induced insight. We propose that (1) the mPFC not only responds to schema-congruent information, but also to the detection of novel schemata, and (2) that the hippocampus responds to a form of associative novelty that is not just a novel constellation of familiar items, but rather comprises a novel meaningful relationship between the items—which was the only difference between our insight and no insight conditions. To investigate episodic long-term memory encoding, we compared CRA items whose solution word was recognized 24 h after encoding to those with forgotten solutions. We found activation in the left striatum and parts of the left amygdala, pointing to a potential role of brain reward circuitry in the encoding of the solution words. We propose that learning from induced insight mainly relies on the amygdala evaluating the internal value (as an affective evaluation) of the suddenly comprehended information, and striatum-dependent reward-based learning.

## Introduction

Insight has been an important subject of investigation in the field of Cognitive Psychology for around a 100 years (Mayer, 1995). By insight, we refer to the phenomenon that sometimes the solution to a previously unsolvable problem is comprehended suddenly as opposed to gradually, usually accompanied by a positive feeling, while being convinced of the correctness of the solution. Several studies suggest that insight can enhance long-term memory (LTM) encoding (Auble et al., 1979; Dominowski and Buyer, 2000; Ash et al., 2012; Danek et al., 2013; Kizilirmak et al., 2015). However, the neural mechanisms that mediate this link between insight and successful encoding are largely unknown. Previous studies suggest that the positive emotional response to insight may play an important role, because successful encoding of an insight solution is associated with higher activation of the amygdala (Ludmer et al., 2011). The hippocampus is critically important for the neural manifestation of explicit memory, and its role in memory includes the detection and encoding of novel stimuli, contexts, and associations (Ranganath and Rainer, 2003). Notably, the hippocampus has also been shown to be involved in the processing of insights (Luo and Niki, 2003), which may provide a further explanation for the facilitated LTM encoding of insight-related information. The aim of the current study is to identify neural correlates of successful encoding of insight solutions, that is, suddenly comprehended presented solutions, via functional magnetic resonance imaging (fMRI) and to stimulate further research by proposing a theory of a neural network involved in learning from induced insight.

When investigating insight, it is important to be aware of the fact that the operationalization of “insight” varies considerably between studies. These variations can be boiled down to two main operationalizations: a relatively objective one, in which the experimenter classifies given problems as either insight or no-insight problems in advance (Auble et al., 1979; Metcalfe, 1986; Bowden and Jung-Beeman, 1998; Wills et al., 2000), and a subjective one, in which participants classify their solution either as being conceived via insight or not after they solved the problem (Bowden and Jung-Beeman, 2003b; Danek et al., 2013, 2014; Kizilirmak et al., 2016). In the experimenter-based approach, insight problems are designed to make it very difficult to solve them gradually by incorporating problem features that usually lead to initial solution attempts, which in turn lead to a dead end, necessitating “thinking outside of the box” to break the fixation on the dead end solution attempt (Öllinger et al., 2014). For example, when one needs to find a common association for words that are only remotely associated, such as “tennis, manners, and cloth” (Bowden and Jung-Beeman, 2003b), one may become fixated on the close associations of the single words such as tennis—ball, racket, player, match, and manners—to say thank-you, holding the door open, gentlemen. Solving this task is very difficult to problem solvers, because it is difficult to think of more remote associations. However, this is necessary to solve the problem (the solution is “table”: table tennis, table manners, and table cloth). In the participant-based approach, insight is assessed by asking participants whether they had an “aha!” experience during the solution of the problem. The subjective “aha!” experience is usually defined as the feeling that the solution was comprehended suddenly, while feeling surprised and convinced of the correctness of the solution. Moreover, once comprehended, the solution appears to be very easy to understand. A few studies suggest that the subjective “aha!” experience does not depend on solving the problem, but that it can also be perceived when confronted with the solution after having unsuccessfully attempted to solve the problem (Bowden and Jung-Beeman, 2003a; Kizilirmak et al., 2015, 2016). It should be noted that both approaches to investigate insight, the experimenter-based classification of insight and no-insight problems, as well as the participant-based classification of solutions accompanied by a feeling of “aha!” (insight) and no “aha!” (no insight), are equally important to gain a better understanding of the mechanisms behind insight. Evidence exists that both cognitive and neural processing differ considerably, when comparing insight and no insight with either approach (Auble et al., 1979; Jung-Beeman et al., 2004; Ludmer et al., 2011; Danek et al., 2014). Both approaches have their own merits: While the number of “insight” and “no-insight” items as well as in which trial and time point “insight” occurs can be controlled in the experimenter-based approach, only the participant-based approach provides information as to whether the participant actually consciously perceives a qualitative difference between insight (“aha!”) and no insight (no “aha!”). We therefore intended to combine both approaches for the current study.

Traditionally, the problems used to study insight are tasks with only one trial, such as the 9-dot problem (Maier, 1930) or the widely known problem with the candle, book of matches, and box of thumbtacks (Duncker, 1945). Although such tasks are well-suited for studying behavioral manifestations or the subjective phenomenology of the insight experience, different tasks are necessary to study the neural underpinnings of insight. Measures of underlying neural activity require multiple measurements of the same kind, that is, many insight problems with minimal differences that all engage comparable cognitive processing strategies. One such task is the CRAT, developed by Bowden and Jung-Beeman (2003b), which is based on the Remote Associates Task by Mednick (1962), originally developed to study creativity. For each trial of this task, a triad of three words is presented which seem completely unrelated at a first glance (e.g., “death, drain, and stem”). Participants are required to find a fourth word that allows (by using this word as a pre- or suffix) to create a compound word with each of the three initially presented words (here: brain, i.e., brain death, brain drain, and brain stem). The CRAT is especially suited for fMRI studies, as a large number of such triads and solutions can be generated, and the solution can be presented to induce insight (i.e., sudden comprehension) at a defined moment in time, after participants had the opportunity to think about the solution for a short time. This also facilitates fMRI data analysis as the variation between participants and trials is relatively small.

For the current study, we used a modified German version of the CRAT where not only solvable (true CRA), but also unsolvable (control) items were presented. Solutions were presented after a short while [4 s presentation of the riddle + 2–8 s fMRI jitter (fixation cross)] to induce insight (sudden comprehension) or not (continued incomprehension) at a well-defined time point. Unsolvable items were created by shuﬄing the triad and solution words of a subset of originally solvable items that was equal in solution rate (when given 30 s to solve an item), probability of experiencing a subjective “aha!” as defined above, and probability to be rated as plausible, based on a prior normative study which assessed these features. Which of four subsets of items was used to create unsolvable items was counterbalanced across participants. This procedure ensured that all differences between the solvable insight condition and the unsolvable control condition could be attributed to sudden comprehension vs. continued incomprehension and not to item-related differences (e.g., word length, frequency, or any other perceptual differences between items). To avoid any misconceptions, we would like to point out that in the current study, we investigated induced insight, that is sudden comprehension following a state of incomprehension induced by presenting the solution, as did Ludmer et al. (2011). This is important to note, because many recent studies operationalized insight as “generating the correct solution to a problem accompanied by a feeling of ‘aha!”’ (e.g., Bowden and Jung-Beeman, 2003b; Jung-Beeman et al., 2004; Danek et al., 2013).

To investigate the neural correlates of successful encoding into LTM, learning trials are usually compared based on whether the encoded items were later successfully remembered or not. Such contrasts are often referred to as “difference due to memory” or DM contrasts, for short (Paller et al., 1987). Importantly, neural correlates of LTM formation are not only determined by the encoding task, but also by the memory retrieval task used to test for encoding success. Depending on how memory is tested, the DM contrast can reflect the encoding of different aspects of the encoded information. In the current study, we used the modified CRAT described above as an encoding task. Participants were not informed that their memory would later be tested, thus, successful encoding was incidental (as opposed to intentional; see Richardson-Klavehn, 2010). The information in the focus of the encoding process during this task may be subdivided into several aspects, namely the triad, the problem, the association between the triad and the problem, as well as episode-specific aspects such as how participants felt when they suddenly comprehended a solution. Memory was tested 24 h later by presenting solution words without their triads, which were either old (i.e., presented during the learning phase) or were new. The task was to decide whether a given solution word had been presented during the encoding task (“old” or “new”). Although recollecting the associated triad or any other contextual information about the encoding episode would almost certainly be helpful during the decision whether a solution was old or new, it was not a necessary requirement for the task. Thus, contrasting learning trials of later recognized and later forgotten solutions should primarily reflect successful encoding of the solution. If successful encoding of the solution were mainly independent of whether the presented solution was comprehended suddenly or not, one would expect no difference between the successful encoding of a CRA or control item’s solution. However, if induced insight, that is, sudden comprehension, facilitated encoding, as we hypothesized, CRA solutions would be expected to be associated with higher recognition memory, higher recollection rates, and differences in neural correlates of successful encoding.

While a number of previous studies investigated the neural correlates of insight, only very few studies have addressed the relationship between the occurrence of an insight and episodic memory at a neural level. Insight as compared with no insight (with differing operationalizations) has been associated with increased activations of the medial temporal lobe (MTL) memory system (right hippocampus, and bilateral amygdala/parahippocampal gyrus) as well as prefrontal brain structures (bilateral inferior frontal gyrus, IFG, middle frontal gyrus, MFG), of the salience network [right insula, right anterior to dorsal cingulate cortex (ACC)], and a temporo-parietal network including the precuneus, the bilateral angular gyrus (ANG), the right superior temporal gyrus (STG), and the right temporal pole (Jung-Beeman et al., 2004; Aziz-Zadeh et al., 2009—also using the CRAT; Luo and Niki, 2003; Qiu et al., 2010)^1^. Brain areas implicated in the processing of insight solutions were the right anterior STG, which may reflect the integration of information across distant semantic relations (Jung-Beeman et al., 2004), the hippocampus, which has been linked to the formation of novel associations (Luo and Niki, 2003), and IFG and ACC, which have been associated with more meta-cognitive processes controlling the search and evaluation of (potential) solutions (Aziz-Zadeh et al., 2009). Sandkühler and Bhattacharya (2008), who also used a version of the CRAT, further suggest that the right temporal activation may reflect retrieval of the novel solution.

Regarding episodic memory for presented insight solutions, analyzed by comparing later recognized old solutions with later forgotten old solutions, the amygdala has been proposed to play an important role due to the positive emotional response in response to sudden comprehension (Ludmer et al., 2011). These researchers further reported the left medial prefrontal cortex (mPFC), ACC, and precuneus from the same contrast (Ludmer et al., 2011). The precuneus is a region which previously has been associated with successful episodic memory retrieval (Shallice et al., 1994; Miller and D’Esposito, 2012) as well as effortful semantic integration (Hagoort et al., 2009; Shimamura, 2011; Seghier, 2013). This may reflect the phenomenon that a solution that could better be semantically integrated was more likely to be remembered later on. The mPFC has recently been suggested to play an important role in the detection and encoding of schema-consistent information, that is, information which can be easily integrated into pre-existing knowledge (Tse et al., 2011; van Kesteren et al., 2012). In this context, insight could also be understood as the rapid formation of a novel schema (Mayer, 1995). Thus, the sudden formation of a novel schema may further support learning from insight.

In short, here, we investigated induced insight and the successful episodic encoding of insight solutions by using a version of the CRAT. To this end, we compared behavioral and fMRI responses to solvable (CRA = induced insight condition) vs. unsolvable items (control condition) and further contrasted later recognized with later forgotten solution words. We hypothesized that induced insight would facilitate encoding via

(a) The positive feeling, which may function as an intrinsic reward and thereby enhance encoding by activating the mesolimbic reward system and
(b) Better semantic integration due to the formation of novel schemata, facilitating the integration of the new information into existing knowledge.

Accordingly, we further hypothesized that, at a neural level, insight-based encoding would engage brain regions previously associated with reward-based learning such as the ventral and dorsal striatum (nucleus accumbens/caudate) (Ikemoto and Panksepp, 1999; Haruno et al., 2004) as well as brain structures previously implicated in schema-based memory formation, most prominently the mPFC (Tse et al., 2011; van Kesteren et al., 2012). We used an episodic recognition memory test, because such tests have often been used to study the influence of reward-related areas on hippocampus-dependent encoding (Wittmann et al., 2005; Krebs et al., 2009a,b; Chowdhury et al., 2012). With respect to the hippocampus we predicted that activations would primarily relate to the detection of novel relationships (Davachi, 2006), rather than successful schema-consistent encoding, which has previously been demonstrate to bypass the hippocampus (Tse et al., 2007; van Kesteren et al., 2013).

## Materials and Methods

### Participants

Twenty-eight graduate and undergraduate students volunteered to participate in our study. Two participants were excluded due to illness or technical problems during scanning. The remaining 26 participants (15 male, 11 female) had an average age of 25 years (*SD* = 3.7, range = 18 to 32 years) and were German native speakers with normal or corrected-to-normal vision. All participants gave written informed consent to participate in the study. At the end of the study, they received financial compensation, and the purpose of the study was explained if requested. The study was approved by the Ethics Committee of the University of Magdeburg, Faculty of Medicine, and was conducted in accordance with the Declaration of Helsinki.

### Material

**FIGURE 1:**
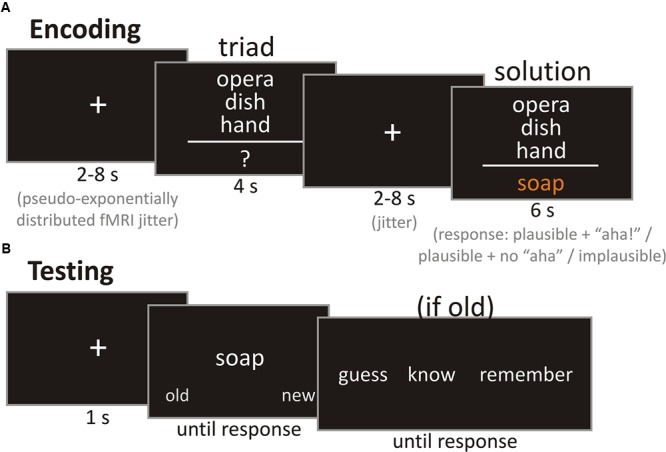
**Example trials of the encoding and test phases. (A)** Exemplary trial of the encoding phase. Participants were asked to press a button depending on whether they thought the solution was plausible while experiencing an “aha!,” plausible without experiencing an “aha!,” or implausible during the presentation of the solution. **(B)** Exemplary trial of the test phase. Participants were asked to decide whether a presented solution was old or new. If an item was judged as old, participants should further differentiate between know and remember, or, if not sure about the solution being old after all, guess.

We used our own German version of the CRAT (Kizilirmak et al., 2016), which is based on the version published by Bowden and Jung-Beeman (2003b) and contains 180 items. Each item consists of three clue words (triad) and a solution word that can be used to form a compound word with each of the triad words (**Figure 1**). The solution word could either be used as a prefix or suffix to build a compound with the other words. Whether the same compound rule (only prefix/suffix or mixed) could be applied to all triad words varied; about half of the items were mixed. All words (triad words and solutions) were nouns or color words. Solution words were only presented in singular form. Due to the German grammatical rules regarding the formation of compound words, some solution words had to be slightly altered (for example by appending an ‘s’) to combine them with the triad words. Solution words were unique while some triad words could appear in up to two different triads.

The resulting normative data were used to divide the 180 items into six pools of 30 CRA items that had approximately equal means with regard to item difficulty, plausibility, and ‘aha’ ratings obtained in a previously conducted normative behavioral study^2^. Two of these pools were used as solvable CRA items and the other two pools as unsolvable control items presented in the encoding phase and the memory test. The remaining two lists were used to provide new solutions for the memory test to provide information about the false alarm rate (new solutions incorrectly categorized as old). Assignment of pools to conditions was counterbalanced across participants by means of a reduced Latin square, such that each pool was used in the CRA, control, and new conditions. This procedure, along with the careful matching of item-pools, ensured that old and new items at test had highly similar normative properties (e.g., *a priori* solution probability).

Unsolvable items for the control condition were created by taking all triad words and solution words of each pool and shuﬄing them separately (i.e., triad words among triad words and solution words among solution words), using the random permutation algorithm from MATLAB 7.1 (The MathWorks, Inc., Natick, MA, USA). The resulting control items were manually inspected for accidental plausibility, meaning that words of an item were semantically associated or could incidentally still be combined to create compound words. In those cases, shuﬄing was repeated until the triad and solution words could no longer be combined. This way, six matched pools of 30 items were created, each composed of four words, in which the triad words could not be combined in a meaningful way with each other or the fourth “solution” word. When an item-pool fell into the control condition according to the counterbalancing scheme just described, the shuﬄed version of that item-pool was used. Owing to the counterbalancing scheme and this shuﬄing procedure, differences between the CRA and control condition could only be due to differences in cognitive processing, and not to differences in perceptual, semantic, or affective properties between individual words, or differences in word frequency in the language.

Four items from the CRA and control conditions each were used in practice trials during the encoding phase. The remaining items were assigned to the two functional MRI runs in equal proportions (56 items per run, 28 items per condition per run).

### Design

The solvable CRA and unsolvable control conditions were presented in event-related manner. For the analysis, the items were further split into conditions according to the participants’ responses. During the encoding phase in the scanner, participants were asked to decide whether a presented solution was plausible, and, if so, whether they experienced a feeling of “aha!” or not, or whether a solution was implausible, once it was presented. During the test phase, participants were presented with new and old solution words and asked to (1) decide whether the solution word was old or new and, if old, (2) whether they remembered something from the encoding context (be it remembering what they thought when they saw the target word, remembering some of the triad words associated with it, etc.), whether they knew due to a feeling of familiarity that the item was old, or whether they were actually not at all sure and simply guessed it was old (Gardiner and Richardson-Klavehn, 2000; Yonelinas et al., 2005). This was done to get information about the quality of the participants’ memory.

### Procedure

#### Encoding Phase

The encoding of the stimuli was performed while participants underwent fMRI scanning at 3 Tesla. Before entering the scanner, participants first ran through a training session with four items from the control and CRA conditions each. This was done to ensure that they understood their task correctly. During scanning, they saw 56 CRA items and 56 control items equally split into two runs. An exemplary trial is shown in **Figure 1A**. Each trial began with a fixation cross which was presented for a duration between 2 and 8 s (pseudo-exponential distribution) which was followed by the triad, presented for 4 s. Participants were instructed to try to think of a solution during that time. From the normative study we knew that only 7% of the CRA items are usually (median solution rate) solved under 6 s. After the triad, another fixation cross was displayed for a variable delay of 2 to 8 s. Thus, with the median duration of the fixation cross jitter following the triad being 4 s and the triad not being displayed during that time, we approximated that probably less than 10% of all items could be solved during that time. The fixation cross was followed by the target word, which was presented for 6 s. Participants were instructed to provide one of three responses during the presentation of the solution: plausible with “aha!,” plausible without “aha!,” and implausible. Plausible with “aha!” and plausible without “aha!” were assigned to either the left or right index finger on a response box (counterbalanced across participants), while implausible required a bimanual response. The definition of the “aha!” experience was an adaptation of the definition provided by Topolinski and Reber (2010) and highlighted that comprehension of the solution should be sudden and unexpected, and that the solution appears to be crystal clear, once it is understood. The description was adapted to be applicable to all solutions, whether they were presented or found by the participant. The “aha!” definition (rough English translation of the German original, cf. Supplementary) read as follows: “You have most likely already had an ‘aha!’ experience yourself. These are moments in which you surprisingly find the solution for a previously incomprehensible problem. You are often unsure how you came up with this solution, but you are convinced of its truth. However, you cannot only experience such an ‘aha!’ when coming up with a solution on your own, but also when you are provided with the solution after you have unsuccessfully thought about the problem on your own. For example, a friend tells a joke which you do not get. He then explains the missing piece of information, and suddenly it all makes sense and you may even ask yourself why you did not comprehend it immediately. In our experiment, the ‘aha!’ experiences may qualitatively diverge from your real-life experiences. It is therefore important to know the following characteristics of an ‘aha!’ experience to make a decision during the task: (1) The solution to the verbal riddle is comprehended **suddenly** and with surprise. (2) The solution, once understood, is comprehended with **ease** and seems very clear. (3) You are **convinced of the correctness** of the solution and do not need to question it. (4) The sudden comprehension is often associated with a **positive feeling**. Importantly, we are not referring to pride, but to the positive feeling which is based on the dissolved tension upon comprehension.

#### Test Phase

Retrieval took place outside of the scanner, approximately 24 h (mean = 24.24 h, *SD* = 1.08 h, range: 22.75–27.67 h) after encoding. During the test phase (see **Figure 1B** for an exemplary trial), participants were presented with solution words from the encoding phase randomly intermixed with new solution words (solution words from CRA items not presented at encoding). Solution words from the eight practice trials were not presented. Presentation of a solution word was preceded by a fixation cross displayed for 1000 ms. Solution words were presented until a response was made. During the presentation of each solution word, participants were to decide whether the word was either “old” or “new” (left or right cursor buttons, counterbalanced across participants). They were specifically instructed that they should only choose “old” when they were sure that the word was seen during the encoding phase the day before in the scanner. This served to split items for the fMRI analysis into later recognized and later not recognized to investigate brain processes during successful encoding (DM effect). When a participant rated a word as “old,” they were further asked to decide whether they *remembered* it and could recollect contextual information or if they only *knew* that the word was old on the next screen (displayed until a response was made). In case “old” was chosen although the participant did not feel confident about the item being old, they should respond with “guess” instead of “know” or “remember.” The remember/know/guess differentiation is a standard procedure to differentiate between familiarity (e.g., recognizing a person as someone who you know, but not knowing who it is or where you know him from) and recollection (e.g., remembering that this is Paul who was sitting in the row in front of you during your last lecture). Please see Gardiner and Richardson-Klavehn (2000) or Yonelinas et al. (2005) for further information and our Supplementary Material for a decision tree for the remember/know/guess/new decision provided as part of the test phase’s instruction sheet.

### Image Acquisition

Scanning sessions were conducted with a 3 Tesla Siemens Magnetom Prisma Syngo MR D13D at the University Hospital of Magdeburg, Germany, with a 64 channel head coil. The MRI session consisted of two anatomical and two functional runs. The first image acquired was a non-distortion corrected T1-weighted image with a resolution of 1.1 × mm 1.1 mm × 7 mm that served as a localizer to set orientation for the following anatomical scan [MP-RAGE sequence, resolution of 1 mm × 1 mm × 1 mm, field of view (FOV) = 256 mm^3^, 192 slices, time to repetition (TR) = 2500 ms, time to echo (TE) = 2.82 ms, flip-angle = 7°], which was used for co-registration of the subsequently acquired functional images. During the two functional MRI runs, blood oxygen level-dependent (BOLD) signal-sensitive T2^∗^-weighted echo-planar images (EPIs) were acquired (voxel size = 2 mm × 2 mm × 3 mm including 10% inter-slice gap; FOV = 216 mm^3^; 34 axial slices aligned to the AC-PC line; TR = 2000 ms, TE = 30 ms, flip angle = 90°). EPIs covered most parts of the brain except for the most dorsal parts of the parietal lobe, sensory and motor cortices. Both functional runs contained 500 scans.

### Image Analysis

Data pre-processing and analysis was done in FSL 5.0 FMRIB’s Software Library^3^ (Smith et al., 2004). Anatomical data were processed with FSL’s brain extraction tool (Smith, 2002), to free cerebral tissue from skull. The functional images were first motion-corrected with the aid of FSL-tool MCFLIRT (Motion Correction FMRIB’s Linear Registration Tool; Jenkinson et al., 2002), followed by slice-time-correction as integrated in FEAT which uses (Hanning-windowed) Sinc interpolation to shift each time-series by an appropriate fraction of the TR relative to the middle of the TR period. EPIs were then smoothed with a full width at half maximum Gaussian kernel of 6 mm. To remove low-frequency signal drifts, a high-pass filter with a cut-off at 100 s was applied to the data. Participants’ functional scans were co-registered with their brain-extracted anatomical scans using FSL FLIRT (FMRIB’s Linear Registration Tool; Jenkinson and Smith, 2001) and spatially transformed into the Montreal Neurological Institute (MNI) standard reference frame.

First level (single-subject) analyses were carried out with multiple regression (parameter estimation via least squares method). Statistical time series analysis was performed using FILM (FMRIB’s Improved Linear Model; Woolrich et al., 2001) implemented in FSL, which includes a local correction of autocorrelations. Two different general linear models (GLMs) were generated: One model contrasted CRA and control conditions, and the other was used to contrast later recognized with later forgotten CRA solutions. Because control solutions yielded too few recognized items, recognized and not recognized items could not be modeled separately, but were collapsed for the control condition. As it could not be assumed that participants would stop trying to solve the triads or think about their solutions before stimulus-offset we used a stimulus-convolved approach to compare CRA and control processing. To this end, we included the presentation times of the triads (4 s) and the (pseudo-)solutions (6 s) for both conditions as predictors in our first GLM. Owing to the concern that conditions with ambiguous responses, that is, “implausible” for CRA and “plausible” for the control condition, may introduce additional variance, we computed the GLM also with additional regressors for the triad and target interval for those “unfitting” combinations (hence called ambiguous trials). While there were too few trials to model separate regressors for CRA + implausible and control + plausible (zero trials in at least one of these conditions in 16 subjects), the composite regressor would nevertheless capture the variance explained by ambiguous trials. The results of the GLM with and without the regressor modeling ambiguous trials were nearly identical. We report the data of the first GLM with the regressor for ambiguous trials included.

In the second GLM, the CRA condition was further split into later recognized and later not recognized items. Because the number of trials in each category was already rather low, we did not model ambiguous items (here: CRA items judged as implausible) in a separate regressor in this model. Just like Jung-Beeman et al. (2004), who also investigated neural correlates of insight with the CRAT, we modeled the presentation of the solution, meaning the moment of deciding the response, as response-locked starting 2 s before the response and ending 2 s after it (4 s interval). In modeling this regressor, it was irrelevant whether the 2 s after button press were already part of the presentation of the fixation cross that followed the presentation of the solution. The rationale behind this approach was to capture the moment of comprehension vs. deciding that the solution is not meaningful, especially as the BOLD response is slow. In both GLMs, all regressors were convolved with a gamma model of the hemodynamic response function, and temporal derivations were added to the model. Functional analysis was done with *z*-statistics, which had been corrected at cluster level according to random field theory. Unless otherwise stated, *z*-threshold was 2.8 and cluster significance threshold was 0.05 (Worsley, 2001).

Due to a programming error, the CRA and control trials of the second run were not presented in a randomized but block-wise manner (first, all solvable CRA items were presented and then all unsolvable control items). Hence, all data were analyzed for both runs separately and not collapsed (28 trials per condition per run). The data from the second run are reported in the Supplementary, as a block-wise presentation of first the CRA and then the control items may have led to different effects. All data reported below are from the first run in which conditions were presented in a randomized order, that is, in an event-related design. Although the results from the first and second runs were overall comparable with respect to the behavioral data and also for the fMRI contrast between CRA and control (seeNeural Correlates of Induced Insight vs. Control here and Test Phase 24 h Later of the Supplementary Materials), the results did differ for the DM contrast (see Neural Correlates of Learning from Induced Insight here and Test Phase 24 h Later of the Supplementary Materials). This suggests that the blocked presentation had an influence on LTM encoding, at least on the neural level.

## Results

### Behavioral Data

#### Encoding Phase

**FIGURE 2:**
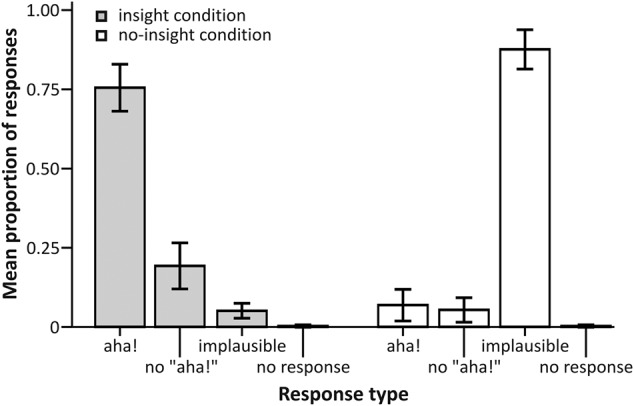
**Proportion of responses for each experimental condition.** Error bars represent 95% confidence intervals.

First, we analyzed the distribution of responses across solvable CRA and unsolvable control items on a purely descriptive level (see **Figure 2** for an overview). A total of 0.75 (*SD* = 0.18) of all CRA items were rated as “plausible” and accompanied by an “aha!” experience, 0.19 (*SD* = 0.18) were rated as “plausible” without being accompanied by an “aha!” experience. Only 0.05 (*SD* = 0.06) of all CRA items were rated as implausible, and participants failed to respond before the start of the next trial in less than 0.01 (*SD* = 0.01) of all CRAT trials. With respect to control items, the majority of items were rated as “implausible” with 0.88 (*SD* = 0.15), while 0.07 (*SD* = 0.12) were judged as “plausible” with “aha!” and 0.05 (*SD* = 0.09) as “plausible” without “aha!.” Again, in less than 0.01 (*SD* = 0.01) of all control items, participants failed to respond.

The high proportion of “plausible” responses for CRA items confirmed a successful induced insight manipulation (seeing an item as “implausible” would preclude sudden comprehension, hence insight), whereas the high proportion of implausible responses for control items corroborates the control condition as a successful no-insight (continued incomprehension) manipulation. Because many response categories contained only very few trials (e.g., no “aha!”| CRA, no response| CRA, implausible| CRA, “aha!”control), we did not split trials according to the participants’ responses for the analysis of the behavioral nor the fMRI data.

For the analysis of response times (RT), median RTs were calculated on an individual level and were then averaged across participants for each condition. For the CRA condition, mean RT was 3453 ms (*SD* = 695 ms) and for the control condition it was 4465 ms (*SD* = 1219 ms). A paired-samples *t*-test confirmed the statistical difference of mean RTs [*t*(25) = 3.68, *p* = 0.001, Cohen’s *d* = 0.748^4^].

#### Test Phase

We analyzed memory performance with respect to the CRA and control conditions. For these analyses, the conditions were not further split depending on the response categories (i.e., plausible with “aha!” / plausible without “aha!” / implausible). All means and standard deviations are reported in **Table 1**.

**Table 1:** Memory performance (proportion of responses) for Run 1 during the test phase 24 h after the encoding phase.

	Insight (CRAT)		No-insight (control)	
	Mean	*SD*	Mean	*SD*
Hit	0.48	0.16	0.39	0.17
Remember	0.20	0.16	0.10	0.09
Know	0.29	0.11	0.29	0.13
Guess	0.06	0.07	0.06	0.09
Miss	0.46	0.17	0.55	0.19

**FIGURE 3:**
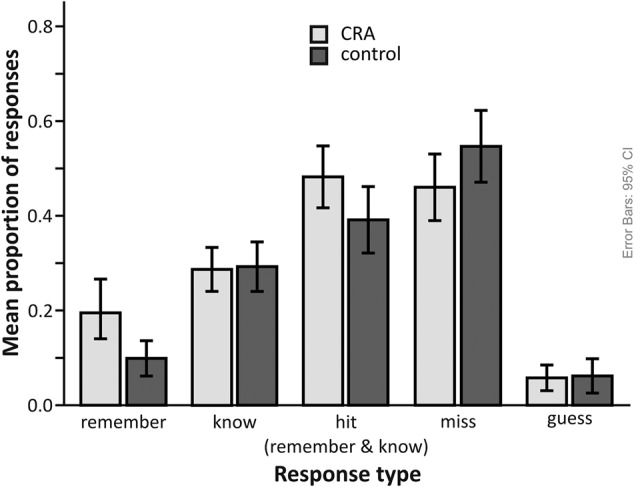
**Recognition memory performance (proportion of responses) for CRA (induced insight) and control items.** The data are split for response category (remember, know, guess, miss). To provide an overview, all responses considered hits, i.e., correctly recognizing items as old, are represented in the “hit (remember and know responses)” bar.

Compared to the control condition (*M* = 0.39, *SD* = 0.17), participants correctly recognized more old solutions from the CRA condition (*M* = 0.48, *SD* = 0.16). A paired *t*-test confirmed this difference to be statistically significant [*t*(25) = 4.36, *p* < 0.001, Cohen’s *d* = 0.955]. Moreover, significantly more solutions were remembered from the CRA compared to the control condition [*t*(25) = 4.74, *p* < 0.001, Cohen’s *d* = 1.061]. The CRA (*M* = 0.29, *SD* = 0.11) and control (*M* = 0.29, *SD* = 0.13) conditions did no differ in regard to their rate of “know” responses as supported by a repeated-measures *t*-test [*t*(25) = -0.25, *p* = 0.823, Cohen’s *d* = -0.063]. In other words, recognition memory only differs for our CRA and control conditions due to a higher remember rate for CRA (see **Figure 3**). This suggests that CRA solutions leave a more detailed memory trace, enabling participants to recollect some information about the encoding episode.

To ensure that these results did not depend on the few cases in which participants responded to CRA items as “implausible” (mean number of trials = 1.4, *SD* = 1.6) or control items as “plausible” (*M* = 3.0, *SD* = 4.8), we ran these analyses again without those trials. The pattern of the results was basically the same. CRA (*M* = 0.47, *SD* = 0.16) and control conditions (*M* = 0.34, *SD* = 0.15) differed significantly in regard to recognition rates [*t*(25) = 5.83, *p* < 0.001, Cohen’s *d* = 1.335]. CRA and control significantly differed in regard to their rate of “remember” responses [*M* = 0.19, *SD* = 0.15 vs. *M* = 0.09, *SD* = 0.08; *t*(25) = 4.65, *p* < 0.001, Cohen’s *d* = 1.096], but not in regard to their “know” response rates [*M* = 0.28, *SD* = 0.11 vs. *M* = 0.25, *SD* = 0.11; *t*(25) = 1.30, *p* = 0.205, Cohen’s *d* = 0.348].

New items were correctly identified in 0.77 (*SD* = 0.17) of all cases.

### Functional Imaging Data

Due to the low number of no “aha!” CRA trials reported as proportions under the section “Encoding Phase” (in absolute numbers of trials, we had <16 trials in 25 participants, and even <10 trials in 20 participants), we could not model “aha!” and no “aha!” separately for CRA items.

#### Neural Correlates of Induced Insight vs. Control

**FIGURE 4:**
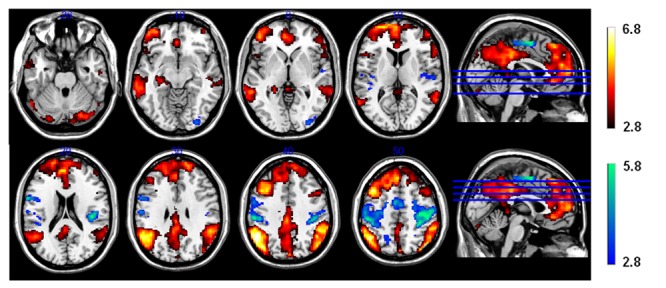
**Functional magnetic resonance imaging (FMRI) contrast for the CRA vs. control condition during the presentation of the solution.** Unfitting responses (i.e., “plausible” for control and “implausible” for CRA) were excluded. White–red activation clusters indicate CRA > control and green-blue activations indicate CRA < control.

The comparison of brain activity during presentation of the triad in the CRA and control conditions revealed no significant differences (*p* > 0.05) in either direction, suggesting similar search processes in both conditions. In other words, participants did not notice whether an item was solvable or not during the presentation of the problem, supporting the comparability of our CRA and control conditions. During the presentation of solution words, however, an increased activation (*Z*-threshold = 3.3, *p* = 0.05) was observed for correct solution words compared to pseudo-solution words (contrast CRA > control) in frontal as well as mediotemporal and inferior parietal regions (**Figure 4**, yellow–red activations). We found higher activation in prefrontal cortical areas, including inferior frontal gyrus, mPFC, and ACC, as well as in the left hippocampus, and in temporo-parietal cortices, including bilateral middle temporal gyrus (MTG), ANG, and supramarginal gyrus (SMG). All activation clusters are summarized in **Table 2**.

**Table 2:** Activation clusters for the insight (solvable CRAT) > no-insight (control) contrast during the presentation of the solution.

Anatomical region	Brodmann area (BA)	Number of voxels	*p*	*Z*	MNI coordinates (mm)		
					*x*	*y*	*z*
1 L middle frontal gyrus;	8, 9, 10, 32, 44, 46	13709	<0.001	6.56	-42 (-14)	18 (43)	52 (26)
L superior frontal gyrus;							
L frontal pole;							
L medial frontal gyrus;							
anterior cingulate cortex;							
2 L angular gyrus;	19, 39, 40	6096	<0.001	7.51	-46 (-51)	-58 (-53)	46 (26)
L supramarginal gyrus, posterior;							
L occipital cortex, superior;							
3 R supramarginal gyrus, posterior;	7, 39, 40	4937	<0.001	6.74	52 (53)	-44 (-50)	44 (29)
R angular gyrus;							
R occipital cortex, superior							
4 L posterior cingulate cortex;	23, 26, 29	4693	<0.001	5.52	-2 (-2)	-32 (-51)	34 (35)
R precuneus							
5 R cerebellum	-	1841	<0.001	5.40	22 (32)	-86 (-75)	-34 (-32)
6 R frontal pole;	45, 46, 48	1293	<0.001	4.54	48 (51)	52 (38)	10 (5)
R inferior frontal gyrus, pars triangularis/pars opercularis
7 L cerebellum	-	1219	<0.001	4.57	-54 (-36)	-58 (-73)	-30 (-33)
8 L hippocampus;	-	334	<0.01	4.26	-28 (-25)	-24 (-29)	-10 (-6)
L parahippocampal gyrus							
9 L middle temporal gyrus;	9	325	<0.01	4.37	-64 (-55)	2 (4)	-28 (-28)
L temporal pole;							
L inferior temporal gyrus							

*MNI coordinates are provided for the peak voxel as well as for the center of activation (in parentheses).*

Calculation of the reverse contrast control > CRA (*Z*-threshold = 3.3, *p* = 0.05) revealed significant activations in brain structures primarily implicated in sensory-motor structures, such as the bilateral sensory-motor cortices (postcentral gyrus and precentral gyrus), and supplementary motor area (SMA) (**Table 3**; **Figure 4** green–blue activations) which is probably due to the high rate of bimanual “implausible” responses for the control condition.

**Table 3:** Activation clusters for the insight (solvable CRAT) < no-insight (control) contrast during the presentation of the solution.

Anatomical region	BA	Number of voxels	*p*	*Z*	MNI coordinates (mm)		
					*x*	*y*	*z*
1 L postcentral gyrus;	2, 3, 4, 6, 43, 48	1885	<0.001	5.24	-48 (-44)	-30 (-20)	46 (48)
L supramarginal gyrus, anterior;							
L precentral gyrus							
2 R postcentral gyrus;	2, 3, 4, 6, 7, 40	1239	<0.001	5.22	36 (38)	-20 (-24)	50 (40)
R precentral gyrus							
3 R supplementary motor cortex;	2, 4	437	<0.001	5.10	0 (-1)	-2 (-8)	56 (54)
R precentral gyrus							
4 R parietal operculum;	48	264	<0.001	4.88	42 (44)	-24 (-23)	20 (20)
R central opercular cortex							
5 L planum temporale;	41, 48	114	<0.05	4.55	-44 (-44)	-34 (-34)	14 (15)
L parietal operculum							

*MNI coordinates are provided for the peak voxel as well as for the center of activation (in parentheses).*

#### Neural Correlates of Learning from Induced Insight

Second, to investigate neural activation during successful episodic encoding of presented CRA solutions (difference due to memory effect, DM-effect), BOLD responses to later recognized vs. later forgotten CRA solution words were compared (*Z*-threshold = 2.3, *p* = 0.05). Because of a relatively low number of remember trials in each run, we compared only hits and misses without further differentiating between remember, know and guess. The results are reported in **Table 4**.

**Table 4:** Activation clusters for the contrast between successfully encoded > not successfully encoded insight solutions.

Anatomical region	BA	Number of voxels	*p*	*Z*	MNI coordinates		
					*x*	*y*	*z*
1 L fusiform gyrus;	19, 20, 21, 22, 37	1045	<0.001	3.62	-42 (-28)	-63 (-78)	-12 (-16)
L middle temporal gyrus;							
L inferior temporal gyrus							
2 L medial frontal gyrus;	44, 45, 48	571	0.008	3.43	-45 (-48)	16 (26)	28 (24)
L inferior frontal gyrus (pars triangularis/pars opercularis);							
3 L superior parietal lobe;	7	430	<0.001	3.43	-30 (-26)	-57 (-54)	50 (52)
L lateral occipital cortex, superior region;							
4 R superior parietal lobe;	7, 40	354	<0.001	3.36	34 (34)	-55 (-54)	47 (50)
R angular gyrus;							
R supramarginal gyrus, posterior region;							
R lateraler occipital cortex, superior region							
5 L caudate nucleus;	-	247	0.01	3.3	-21 (-16)	5 (18)	0 (8)
L putamen;							
L amygdala							
6 Thalamus, medial dorsal and anterior parts;	-	223	0.03	3.02	-5 (-8)	-7 (-2)	11 (12)

*MNI coordinates are provided for the peak voxel as well as for the center of activation (in parentheses). There were no differential activations in the opposite direction for the chosen significance threshold.*

**FIGURE 5:**
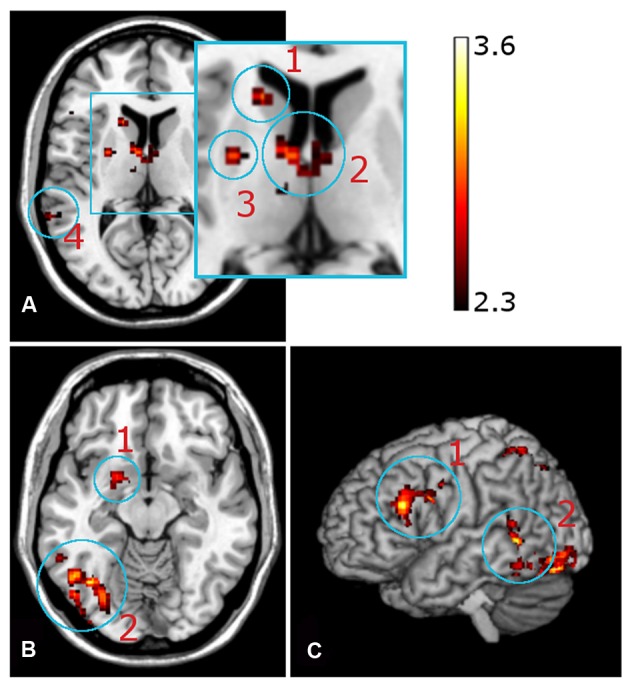
**Functional magnetic resonance imaging contrast for successfully encoded (later recognized) > unsuccessfully encoded (later forgotten) CRA solutions. (A)** Axial view, *z* = 10, (a1) left caudate nucleus, (a2) anterior thalamus, (a3) putamen, (a4) inferior temporal gyrus. **(B)** Axial view, *z* = -12, (1) amygdala, (b2) inferior temporal gyrus. **(C)** Sagittal view. (c1) inferior frontal gyrus, (c2) inferior/medial temporal gyrus.

**FIGURE 6:**
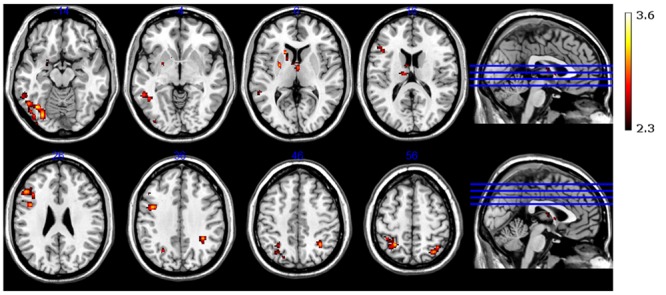
**Functional magnetic resonance imaging contrast for successfully encoded (later recognized) > unsuccessfully encoded (later forgotten) CRA solutions**.

Neural activation was observed in the left amygdala (**Figure 5B**), left putamen and left caudate nucleus (**Figure 5A**), bilaterally in the anterior and dorsomedial thalamus (**Figure 4**), and in the left inferior/middle frontal gyrus (**Figure 5C**). Further activation clusters were observed in temporo-parietal regions, namely within the posterior part of the left inferior temporal gyrus (ITG), and in the inferior parietal lobe (IPL), spanning the right SMG and ANG (**Figure 6**). All activation clusters for the DM-effect are summarized in **Table 4**.

## Discussion

The current study aimed to illuminate the neural correlates of induced insight and successful explicit memory formation for presented insight solutions by comparing solvable CRA (insight) and unsolvable control problems, as well as by contrasting encoding trials of later recognized and later not recognized CRA solutions. We had hypothesized that induced insight, that is, sudden comprehension of a previously incomprehensible problem upon the presentation of the solution, would evoke a positive feeling which may serve as an intrinsic reward, thereby facilitating successful encoding. Though this positive emotional response would probably be considerably weaker compared to generating the solution themselves, most likely due to the missing pride of solving the puzzle, evidence exists that it is still often accompanied by a moderate positive response (Kizilirmak et al., 2016). We had further hypothesized that CRA items would be associated with better semantic integration due to the formation of novel schemata, facilitating the integration of the new information into existing knowledge.

### Induced Insight is Associated with Better Learning of the Solution

Of all solvable CRA items (induced insight condition), almost three quarters were rated to have elicited an “aha!” experience (as described under the section “Encoding Phase”). This finding supports the idea that, even when correct solutions are presented rather than found by the participants themselves, a subjective experience of “aha!” can be induced. On the other hand, unsolvable control items were correctly identified as implausible in almost 90% of all cases. Slightly more than 10% of those items were rated as plausible (either with or without “aha!”), suggesting that participants may, at some instances, have failed to press both buttons for the implausible response simultaneously, as required, or that participants might have occasionally found their own creative individual associations between the triad words.

In line with the assumption that insight facilitates encoding into LTM (Auble et al., 1979; Dominowski and Buyer, 2000; Ash et al., 2012; Danek et al., 2013; Kizilirmak et al., 2015), we observed higher recognition rates for CRA solutions compared to the control condition’s “solutions” as well as higher recollection rates for the CRA solution. The higher recollection rates indicate that memories for CRA solutions were associated with a more elaborate recollective experience, and were in this sense more “episodic” (Gardiner and Richardson-Klavehn, 2000; Yonelinas et al., 2005). In fact, the superior memory performance for CRA items could be almost exclusively attributed to recollection (**Figure 3**). This observation is similar to the commonly reported preferential contribution of deep (i.e., semantic and/or elaborate) study processing to recollection compared with familiarity (Gardiner et al., 1996; Gardiner and Richardson-Klavehn, 2000).

One limitation of the present study is that we did not collect further information with respect to the content of the contextual information recollected, for example, whether participants recollected triad words associated with a solution or what they felt when they saw the solution during encoding. We suggest that the most likely information retrieved would be the triad words associated with the solution, but we cannot exclude that this particular information could also be retrieved when participants correctly recognized the target word based on familiarity. Therefore, we can only speculate that induced insight was most likely associated with higher positive emotional responses (Danek et al., 2013, 2014; Kizilirmak et al., 2015) and better integration of the novel information into pre-existing knowledge (van Kesteren et al., 2012). These potential explanations are supported at a neural level by the higher activation of the amygdala and striatum as well as the mPFC for insight vs. no insight, as discussed below.

### Neural Correlates of Induced Insight and Insight-Related Memory Encoding

Regarding the neural correlates of induced insight vs. control and successful (later recognition of old items) vs. unsuccessful encoding (later misses), it is remarkable that differences were only found for the presentation of the solution but not for the presentation of the problem itself. This suggests (1) that our control condition was not obviously unsolvable when presented without the (pseudo-)solution, but well comparable to the actual remote associations from the CRA condition which also seem not associated at first glance, (2) that the CRA and control items differed only in regard to sudden comprehension vs. continued incomprehension when the solution was processed, and (3) that the relevant encoding processes, which either led to later recognition or non-recognition of the solution, occurred during the processing of the solution.

The increased activations observed for CRA compared to control items were largely consistent with previous findings. In line with the idea that insight reflects the sudden comprehension of a *novel* relationship between the solution word and the triad, we found that insight was associated with a higher activation of the left hippocampus. This activation is compatible with the finding by Luo and Niki (2003). Luo and Niki (2003) interpreted the observed hippocampal activation as reflecting reorienting processes, implying both the breaking of mental fixations on unsuccessful solution attempts as well as the formation of novel associations (Luo and Niki, 2003). The present finding could be explained analogously. Importantly, in the context of the CRAT, neither the triad or solution words nor the compound words *per se* were novel to the participants. Instead, the novelty of the relationship between triad and solution words is a purely associative one, as it is solely defined by the sudden comprehension that the triad words have a common link in the target word. Our data thus conform with our initial hypothesis that the primary role of the hippocampus in insight processing is the detection of novel associations. This is also in line with earlier studies that have more generally implicated the hippocampus in the detection of associative novelty, as defined by a novel combination of familiar items (Düzel et al., 2003; Schott et al., 2004; Davachi, 2006). Because novel combinations of familiar items occurred in both, the solvable and the unsolvable condition, our data extend these findings by further suggesting that the hippocampus may be particularly sensitive to the novel *meaningful* relationships between familiar items.

The induced insight condition differed from the control condition also with respect to prefrontal cortical activations, specifically in the mPFC, both rostral and dorsal ACC and IFG. Similar to the interpretation by Aziz-Zadeh et al. (2009), the ACC and IFG may have been involved in the evaluation of the presented solution (Aziz-Zadeh et al., 2009). The dorsal ACC would most likely act as a salience detector here (Seeley et al., 2007), whereas the rostral ACC would rather be part of the mPFC schema encoding network (van Kesteren et al., 2013). More generally, particularly the left IFG has been implicated in the semantic analysis of verbal information (Demb et al., 1995; Poldrack et al., 1999; Schott et al., 2013; Soch et al., 2016) and also in the retrieval of information from semantic (as opposed to episodic) memory (Düzel et al., 1999). In the present study, IFG activation might constitute a neural correlate of retrieving semantic information regarding the compound words from semantic memory (e.g., by checking with the pre-existing English vocabulary whether “brain” and “death” can be combined to a meaningful known compound word).

The mPFC on the other hand has not yet been implicated in the context of insight as compared to no insight, although it has been reported to be associated with successful encoding of insight solutions (Ludmer et al., 2011). We suggest that the stronger activation of this region for insight as compared to no insight items most likely reflects the processing of schema-congruency. Previous studies have demonstrated that the mPFC is critically involved in the rapid encoding of novel information into pre-existing schemata (van Kesteren et al., 2010, 2012). In those studies, however, participants had acquired a schema prior to the study, and mPFC involvement could therefore only be demonstrated for encoding of novel, but schema-congruent stimuli. Here, on the other hand, providing the solutions to the triad words presented before most likely resulted in the almost instantaneous formation of previously non-existing schemata. We therefore suggest that, in addition to its, by now well-established, role in the encoding of schema-congruent information, the mPFC is also involved in the initial formation of a schema—at least when this occurs at a rapid time scale. In addition to schema congruency, mPFC activation in response to CRA as compared to control solutions might, to some extent, be associated with reflecting on pre-existing semantic associations, which contributes more to deep as compared to shallow memory encoding (Schott et al., 2013).

In contrast to studies by Jung-Beeman and Bowden (2000), Jung-Beeman et al. (2004) and Kounios et al. (2006), we did not find activation in the anterior STG. In the aforementioned studies, this region had been found when contrasting CRA items that were solved and accompanied by a subjective “aha!” “experience with CRA items solved without “aha!” experience. Considering that we compared CRA items collapsed across “aha!”/no “aha!” trials (due to the low number of no “aha!” responses) with unsolvable CRA-like control items, this difference is not surprising. It moreover suggests that there is a neural processing difference between insight and no insight, depending on whether this refers to sudden comprehension vs. continued incomprehension or the subjective experience vs. non-experience of an “aha!,” that is, the feeling that the solution is comprehended suddenly, accompanied by a positive emotional response, being convinced of the correctness of the solution, and feeling that the solution is very clear and easily comprehensible once understood.

The DM contrast revealed that brain regions involved in successful LTM encoding of CRA items overlapped only partially with those involved in the CRA condition *per se*. Specifically, the only robust overlap between induced insight processing and successful encoding of insight solutions was observed in inferior parietal regions (ANG, SMG), which might be explicable by attentional processes (Corbetta and Shulman, 2002; Cabeza et al., 2012). Alternatively, or additionally, the temporo-parietal junction (i.e., ANG and SMG) has also been implicated in level of processing (LOP) during episodic encoding (Schott et al., 2013). Strikingly, whereas in that study, we observed encoding-related functional connectivity increase of the hippocampus with the left IFG, the mPFC (see above) and the TPJ, only the hippocampal-TPJ connectivity increase predicted the degree of the LOP effect at the level of individual participants. Given this somewhat comparable involvement of overlapping brain structures in deep encoding and in the processing and encoding of CRA items, we tentatively suggest that insight-related encoding might, to some extent, reflect a special case of deep (i.e., semantic, associative) encoding.

In line with our hypothesis that encoding of insight-associated information might be related to positive feelings during sudden comprehension, the DM contrast revealed increased activation of the amygdala during successful encoding of presented insight solutions, a finding in line with a previous study by Ludmer et al. (2011). This supports their idea that emotional arousal during processing of the solutions may contribute to successful encoding. Furthermore, successful encoding of CRA solutions was also associated with activations of the striatum, particularly the caudate nucleus, extending into the ventral striatum (**Figure 3**). The role of the ventral striatum in reward processing is a well-replicated finding (Knutson et al., 2001; Wittmann et al., 2005), and activation of more dorsal portions of the caudate has been associated with short-term reward (Haruno et al., 2004) and with reinforcement-based learning (Kahnt et al., 2009). Given the previously reported improved explicit encoding of reward-associated stimuli (Wittmann et al., 2005; Adcock et al., 2006; Krebs et al., 2009a,b), a rather straight-forward explanation for the striatal activation observed in the present study would be the notion that learning from insight may in part be driven by the processing of intrinsically rewarding information, which has also been associated with recruitment of the mesolimbic reward system (Daniel and Pollmann, 2010). Notably, participants did not solve items on their own, but were presented with a solution word (after an interval of generally unsuccessful solution attempts) for which they needed to comprehend how it could be combined with the triad words to build compound words. Thus, even though the rewarding feeling of sudden comprehension was probably lower than one might expect for generated solutions, it seems to have been strong enough to elicit increased striatal activation.

Somewhat surprisingly, neither the hippocampus, nor the mPFC differentiated between successfully vs. unsuccessfully encoded CRA solutions. Although one has to be careful with null effects, one potential explanation for this finding could be the way memory was tested. Memory for the solution was probed via an old/new recognition test. Only the solution was presented, and it was not necessary to retrieve any associated information (e.g., the triad words), in order to decide whether a solution word had been presented 24 h earlier. Such a decision could be achieved solely on the basis of familiarity, although the behavioral results clearly indicate that the recognition memory advantage for CRA solutions could be largely attributed to recollection. We tentatively suggest that the hippocampus might have already been strongly engaged by the encoding of the novel meaningful relationship, irrespective of later recognition (**Figure 4**), such that subtle differences in hippocampal activation might not have been picked up by the DM contrast. Given the recent identification of the differential contribution of hippocampal input and output structures to novelty detection and successful LTM encoding (Maass et al., 2014), we propose that future research should employ high-field fMRI to detect a potential contribution of hippocampal output structures (i.e., pyramidal CA1, deep entorhinal cortex) to successful encoding of insight-associated information.

### Limitations

One limitation of our study is that the number of trials per condition in the DM analysis was rather low, and we were therefore not able to further separately consider ambiguous trials (i.e., CRA trials rated as implausible) in that analysis. It should be noted, on the other hand, that the inclusion of a separate regressor for ambiguous trials did not qualitatively affect the results of our main statistical model (if at all, we observed somewhat larger clusters when including the regressor; see Materials and Methods section for details), and it thus appears that, in our view, it would be unlikely that considering ambiguous trials separately in the DM analysis would substantially affect the results.

Along the same line, it must be acknowledged that the number of subsequently recalled items in the control condition was too low to allow for a DM type analysis. We can therefore not completely exclude the possibility that successful encoding of the control items might engage a comparable network of brain structures. Given the predominant engagement of the hippocampal-prefrontal networks observed in more “classic” DM studies and the previously demonstrated role of the striatum in intrinsic reward (Daniel and Pollmann, 2010), along with the unlikeliness of the control items to elicit intrinsic reward responses, we nevertheless suggest that the involvement of the mesolimbic network in successful encoding is at least to some extent specific to the insight-inducing task used here.

Another limitation concerns the activation of the amygdala and the striatum during insight processing successful encoding. While activation of these brain regions has repeatedly been linked to reward processing and/or emotional arousal, we did not record an objective measure of arousal, such as skin conductance or pupillary dilation in the present study. Such a psychophysiological measure would be of particular interest when comparing “aha!” and “non-aha!” items, and future research should be aimed at differentiating objective and subjective insight manipulations also at the level of psychophysiology.

## Conclusion

The findings of the current study suggest that encoding of solutions to verbal riddles is more successful when the solution is comprehended suddenly (CRA = induced insight) as compared to continued incomprehension (control). We further found that induced insight was associated with higher activation of several frontal, temporal, and parietal brain regions of which we would like to point out the hippocampus and mPFC. The hippocampus has been known to be involved in associative novelty, however, never in the sense of detecting a novel *meaningful* combination of known items (insight condition) as compared to just a novel combination of known items (control condition). Thus, the hippocampus may play a special role during insight processing, by detecting novel meaningful relationships. The mPFC on the other hand has been associated with detecting schema-consistency and may be associated with the detection that a novel meaningful relationship is consistent with existing knowledge. Regarding the neural correlates of successful encoding of CRA items, our current findings suggest that (1) the positive emotional response toward sudden comprehension (insight) as reflected by higher activation of the amygdala and (2) intrinsic reward as reflected by higher activations of the striatum play key roles in learning from insight. Our findings suggest that encoding insight-related information is different from the encoding of non-insight related information, because it seems to rely on reward learning, which is not typical for information that is not associated with external rewards. We would therefore propose that insight, that is, sudden comprehension of a solution, may itself be rewarding, thereby facilitating LTM encoding of insight-related information.

## Author Contributions

AR-K conceived this line of research, and JK and AR-K conceived and designed the experiment. JK and HT developed the CRAT materials and programmed the experiment. BS gave advice on fMRI-related design questions, and contributed significantly to the writing of theoretical sections on neural correlates of LTM formation and dopamine. JK wrote the first draft of the section “Introduction and Discussion” and coordinated the writing of the manuscript. HT conducted the study, wrote the first draft of the section “Materials and Methods,” and analyzed the data. KF-S wrote the first draft of the section “Results.” AR-K, BS, and KF-S funded the study/publication. All authors were critically involved in the interpretation of the results and in revising first versions of the manuscript.

## Conflict of Interest Statement

The authors declare that the research was conducted in the absence of any commercial or financial relationships that could be construed as a potential conflict of interest.

## Footnotes

1 Studies contrasting an insight condition with a null-event baseline were not included in this summary as such a contrast may include unspecific effects and therefore not accurately reflect the differential activation of insight solutions compared to no-insight solutions.

2 The normative data can be requested via e-mail from the corresponding author: jasmin.kizilirmak@scienceforfun.org.

3 http://fsl.fmrib.ox.ac.uk/fsl/fslwiki/

4 Cohen’s *d* was always calculated via *d*_4_ *= M*_D_*/SD*_D_, Cohen’s *d = d*_4_*/√(r)*.
