# Supplementary material for: Neural Correlates of Learning from Induced Insight: A Case for Reward-Based Episodic Encoding
Source: Front Psychol. 2016 Nov 1;7:1693. doi: 10.3389/fpsyg.2016.01693 (PMC5088210; doi:10.3389/fpsyg.2016.01693)
Supplement: Supplementary file 1 [file Presentation_1.PDF]

Supplementary Material to "Neural correlates of learning from induced insight: A case for reward-based encoding"

J. M. Kizilirmak, H. Thuerich, K. Folta-Schoofs, B. H. Schott, & A. Richardson-Klavehn

1 Supplementary methods

1.1 Instructions

The original written instruction, as provided in German, are provided below.

1.1.1 Learning phase in the scanner

„Instruktion

In diesem Experiment wollen wir einerseits "Aha!"-Erlebnisse und andererseits das Lösen von Worträtseln untersuchen. Das Experiment findet an zwei aufeinander folgenden Tagen statt.

Die Aufgabe

In der folgenden Aufgabe muss zu jeweils drei Wörtern ein viertes Wort gefunden werden. Dieses vierte Wort stellt eine Verbindung zwischen den ersten dreien her, indem es sich mit jedem einzelnen von ihnen entweder als Präfix oder Suffix zu einem neuen Substantiv verbinden lässt. Es kann dabei auch sein, dass die Form eines Substantivs leicht abgeändert werden muss, um ein verbundenes Substantiv zu bilden, z.B. "Marmelade" + "Glas" = "Marmeladenglas".

Auf den ersten Blick werden die drei Rätselwörter scheinbar kaum etwas miteinander zu tun haben und es wird dir schwer fallen, einen Zusammenhang herzustellen, z.B. Silber - Essig - Preis. Manchmal wird dir dann "ein Licht aufgehen" – entweder, wenn du selbst auf eine Lösung kommst, oder wenn dir das Lösungswort präsentiert wird – und die Verbindung zwischen den Wörtern ist mit einem Mal ganz klar. In unserem Beispiel wäre das "Tafel" für *Tafel*silber, *Tafel*essig, Preis*tafel*.

Ein Durchgang wird so ablaufen, dass zunächst ein kleines *Fixationskreuz* zu sehen ist.

An dieser Stelle werden dann *drei Wörter* erscheinen. Deine Aufgabe ist es, dir ein Verbindungswort zu überlegen. Meist wird die Zeit zur Lösungssuche recht kurz sein, was die Aufgabe sehr schwierig macht. Wir möchten dich jedoch bitten, immer selbst nach einer Lösung zu suchen, da wir uns gerade für diese Prozesse interessieren. Es wird dir dadurch auch leichter fallen, eine ähnliche Aufgabe bei der nächsten Messung zu bearbeiten.

Nach den Rätselwörtern wird wieder ein Fixationskreuz erscheinen. Bitte nutze auch diese Zeit noch, um nach der Lösung des Rätsels zu suchen.

Danach erscheint ein von uns als sinnvoll eingestuftes *Lösungswort* unter den Rätselwörtern. Versuche bitte den Zusammenhang zwischen dem Lösungswort und den Rätselwörtern zu finden. Sobald du ihn gefunden hast, gib bitte per Tastendruck an, ob du beim

Finden des Zusammenhangs ein Aha-Erlebnis hattest oder nicht. Siehst du keinen Zusammenhang und findest die Lösung unplausibel, drücke bitte beide Tasten gleichzeitig. Welche Tasten du für Aha/kein Aha/unplausibel drücken musst, wird dir noch vom Programm mitgeteilt.

Bitte beachte die folgende Beschreibung, was ein Aha-Erlebnis ist, bei deiner Beurteilung.

Definition eines Aha!-Erlebnisses

Ein Aha-Erlebnis hast du bestimmt schon selbst einmal erlebt. Das sind die Momente, in denen man überraschend zu einer Erkenntnis kommt, d.h. man findet plötzlich die Lösung für ein zunächst nicht oder nur schwer lösbar scheinendes Problem. Oft ist man sich nicht sicher, wie man zu dieser Erkenntnis gelangt ist, jedoch ist man sich ziemlich sicher, dass die gefundene Lösung richtig ist, ohne diese weiter hinterfragen zu müssen. So als ob sie mit einem Mal einfach da und völlig klar ist. Wenn man zum Beispiel für ein in Schule, Beruf, Studium oder im persönlichen Bereich bestehendes Problem nach längerem Nachdenken ganz plötzlich auf eine Lösung kommt. Doch kann man Aha-Erlebnisse nicht nur haben, wenn man selbst auf eine Lösung kommt, sondern auch wenn man die Lösung für etwas vorgegeben bekommt, über das man eine Weile

nachgedacht hat. Zum Beispiel wird einem von einem Freund ein Witz erzählt und man versteht die Pointe nicht sofort. Wenn der Freund einen dann aufklärt, denkt man sich „Ach, ja! Genau!“ und fragt sich eventuell sogar, warum man nicht von selbst auf die Lösung gekommen ist.

In unserem Experiment werden die Aha-Erlebnisse wohl qualitativ etwas von denen in deinem persönlichen Alltag abweichen. Daher ist es wichtig zu wissen, welche Qualitäten für ein Aha-Erlebnis sprechen, wenn du die Rätsel löst: (1) Die Lösung auf das Worträtsel wird dir plötzlich und überraschend klar und (2) die Lösung erscheint dir auf einmal ganz einfach – sie „flutscht“ richtig, so wie wenn man ein Marmeladenglas öffnen möchte und trotz hohem Kraftaufwand tut sich erst einmal nichts. Doch dann, ganz plötzlich, lässt es sich ganz einfach drehen und öffnen. So ähnlich wird es auch in deinem Kopf aussehen. Du denkst über ein Rätsel nach und merkst richtig, wie angespannt du dabei bist. Mit dem Finden oder Verstehen löst sich diese Anspannung und du hast das Gefühl, als würden sich deine Nerven wieder entspannen. Wir würden sagen, die Verarbeitung der Lösung ist flüssig und mühelos. Des Weiteren (3) hast du Vertrauen in die Lösung, d.h. du bist überzeugt von dessen Richtigkeit, ohne dies hinterfragen zu müssen. Das plötzliche Verstehen der Lösung (4) geht auch des Öfteren mit einem positiven Gefühl einher. Hierunter ist nicht das Stolz sein über die eigene Leistung gemeint, sondern ein angenehmes Gefühl, welches durch das Verstehen und der Überzeugung von der Richtigkeit der Lösung sowie durch das Lösen der Anspannung beim Nachdenken, hervorgerufen wird.“

1.1.2 Testing phase 24 hours later

"Instruktion

Heute wirst du eine Liste von Wörtern sehen. Einige dieser Wörter tauchten gestern als Lösungswort im Experiment auf, weiterhin sind auch Wörter enthalten, die gestern nicht während des Experimentes zu sehen waren. Deine Aufgabe wird es sein zu entscheiden, ob es sich bei dem Wort, welches du gerade siehst, um ein altes Wort oder um ein neues Wort handelt. Drücke bitte ALT, nur wenn du sicher bist, das Wort als eines der gestern gesehenen Lösungswörter zu erkennen. (Anmerkung: Es tauchen keine der Hinweiswörter von gestern auf, lediglich die Lösungswörter.) NEU drückst du, wenn du dir nicht sicher bist, ob das Wort gestern dabei war, oder du dir sicher bist, dass es neu ist.

Weiterhin interessiert uns, wie Wörter, die man wiedererkennt im Bewusstsein verankert sind.

Beim Wiedererkennen gibt es zwei verschiedene Arten des Bewusstseins, die unterschieden werden:

Zum einen kann das Wiedererkennen eines Wortes damit verbunden sein, dass einem der Moment wieder ins Bewusstsein kommt, als man das Wort vorher (in diesem Fall gestern) sah. Diese Form des Bewusstseins nennen wir Erinnern. Dies beinhaltet alle Arten des mentalen Wiedererlebens.

Du erinnerst dich zum Beispiel daran,

...in welchem Kontext du dieses Wort gesehen hast (z.B. erinnert man sich an Rätselwörter, die gestern mit diesem Lösungswort zusammen präsentiert wurden, oder andere Lösungswörter, die vorher oder nachher auftauchten).

...was du dabei gedacht hast, als du das Wort sahst, dass es z.B. eine überraschende Lösung für das Problem war. Es könnte auch sein, dass dich das Wort an eine persönliche Episode in deinem eigenen Leben erinnert hat, als du es gestern sahst. Zum Beispiel taucht das Wort "Harfe" auf und du bist dir sicher, dass dieses Wort gestern dabei war, weil du, als du das Wort sahst, daran dachtest, dass du neulich in einem Restaurant warst, in dem zum Essen Harfenmusik gespielt wurde und dir beim Anblick des Wortes dieser Moment in Erinnerung kam.

Es kann aber auch sein, dass du dich einfach nur daran erinnerst, wie du das Wort auf dem Bildschirm gesehen hast, z.B. weil es dir merkwürdig vorkam. Auch die Erinnerung daran, wie man das Wort wahrgenommen hat, ist "erinnern".

Die andere Form bezeichnen wir als Kennen. Hierbei ist man sich sicher, dass jenes Wort gestern vorhanden war, jedoch fehlt die genaue Erinnerung an das Erlebnis selbst, so wie oben beschrieben. Vielmehr handelt es sich um ein starkes Gefühl der Vertrautheit, das Wort gestern gesehen zu haben. Sicherlich kennst du Situationen, in denen dir ein Gesicht einer Person sehr vertraut vorkommt und du weißt, dass du sie in der letzten Zeit gesehen hast, jedoch kannst du keine anderen Informationen zu dieser Person, wie Namen, Ort und Zeit der Begegnung abrufen. So ähnlich gestaltet sich das auch für

diese Form des Bewusstseins in Bezug auf Wörter, bei denen du dir sicher bist, dass ein Wort gestern dabei war, du dich jedoch nicht genau daran erinnern kannst es gesehen zu haben, da jede Art des Wiedererlebens fehlt.

Wenn du nun in der Liste ein Wort siehst, welches du sicher als gestern gesehen wiedererkennst, und dementsprechend ALT drückst, sollst du danach angeben, ob du dich an dieses Wort erinnerst (dafür drücke bitte ERINNERT), oder ob es dir von gestern lediglich stark vertraut vorkommt, da du keine direkten Erinnerungen daran hast, wie du es gesehen hast (dafür drücke bitte ERKANNT). In manchen Fällen kann es sein, dass du, einem ersten Impuls folgend, fehlerhaft bei einem Wort ALT gedrückt hast, du dich aber eigentlich weder genau erinnerst, noch ein starkes Gefühl der Vertrautheit von gestern hast. In diesem Fall drücke bitte GERATEN.

Um das, was du eben gelesen hast, noch einmal zusammenzufassen, haben wir dir hier mal einen Entscheidungsbaum aufgezeichnet, der dir mit Gedankenbeispielen helfen soll, dich zwischen den drei Kategorien ERINNERT, ERKANNT und GERATEN zu entscheiden.

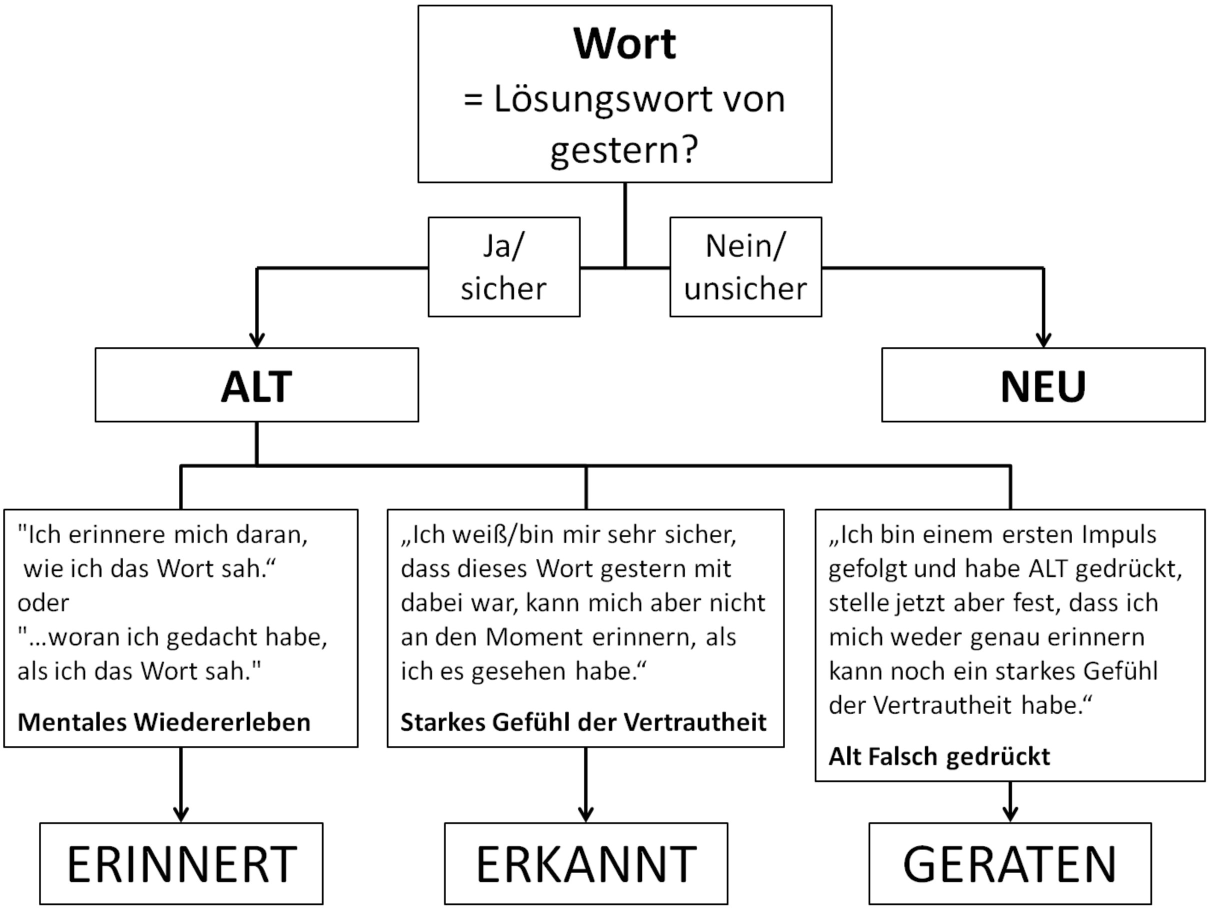

Falls du nun noch Fragen zu deiner Aufgabe haben solltest, kannst du diese gern an den Versuchsleiter richten."

Here is an English translation of the flowchart provided in the instructions of the memory test session to differentiate between remember, know, and guess:

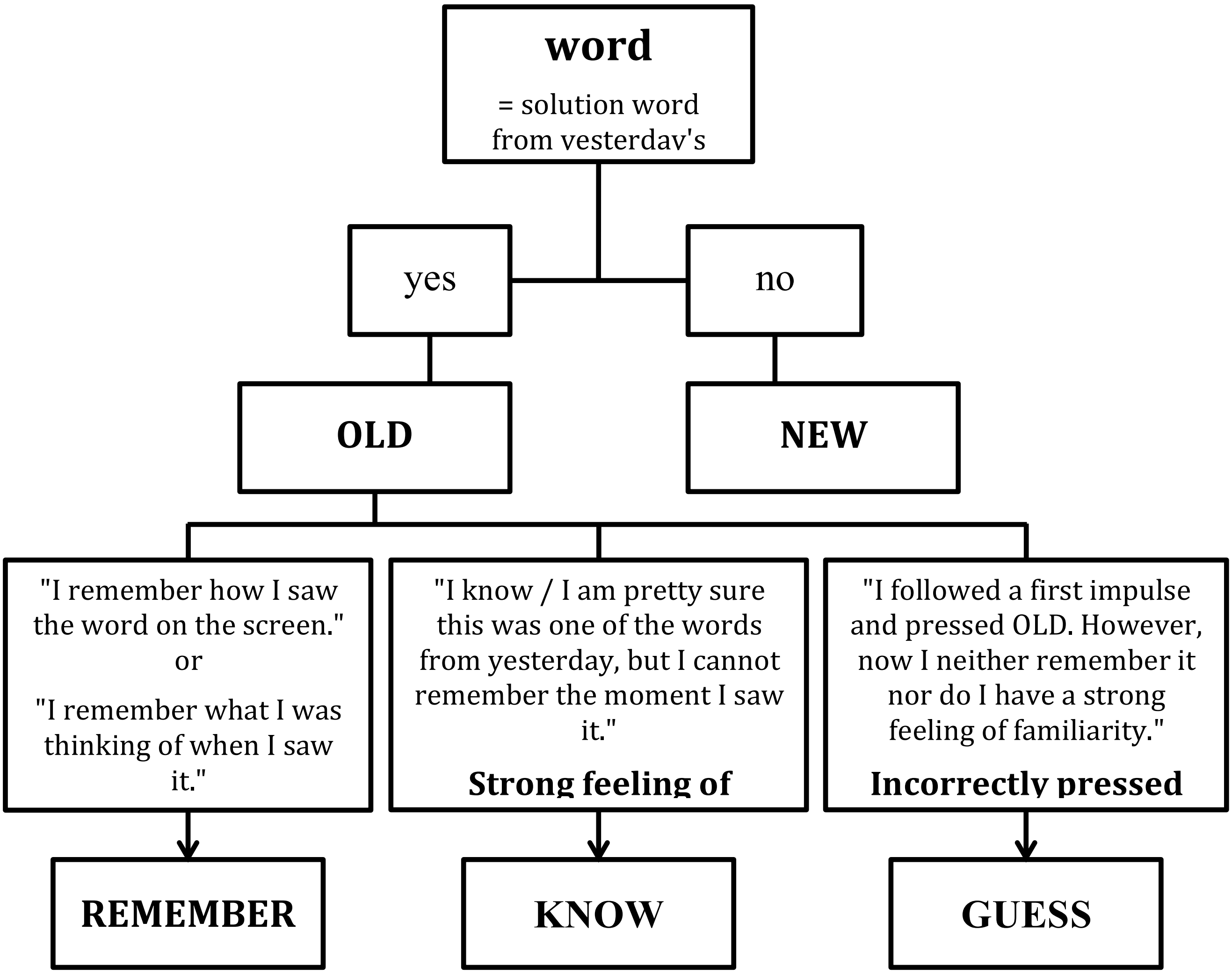

2 Results of the 2nd run

Due to an error in the script of the experimental presentation, the trials of the second run were not presented in a event-related but blocked design with the insight items being presented first and the no-insight items being presented afterwards. Cognitive processing may have been different from the event-related presentation, which is supported by the at least partially differing results of the fMRI contrasts.

2.1 Behavioral data

First, we analyzed the distribution of responses across solvable CRAT (insight) and unsolvable control (no insight) items on a purely descriptive level (see Figure S1). A total of .68 (SD = .23) of all CRAT items were rated as “plausible” and accompanied by an “aha!” experience, .26 (SD = .24) were rated as “plausible” without being accompanied by an “aha!” experience. Only .05 (SD = .05) of all CRAT items were rated as implausible, and participants failed to respond before the start of the next trial in .01 (SD = .06) of all CRAT trials. With respect to control items, the majority of items were rated as “implausible” with .88 (SD = 0.18), while .07 (SD = .15) were judged as “plausible” with “aha!” and .04 (SD =

.08) as “plausible” without “aha!”. Again, in only .02 (SD = .04) of all control items, participants failed to respond.

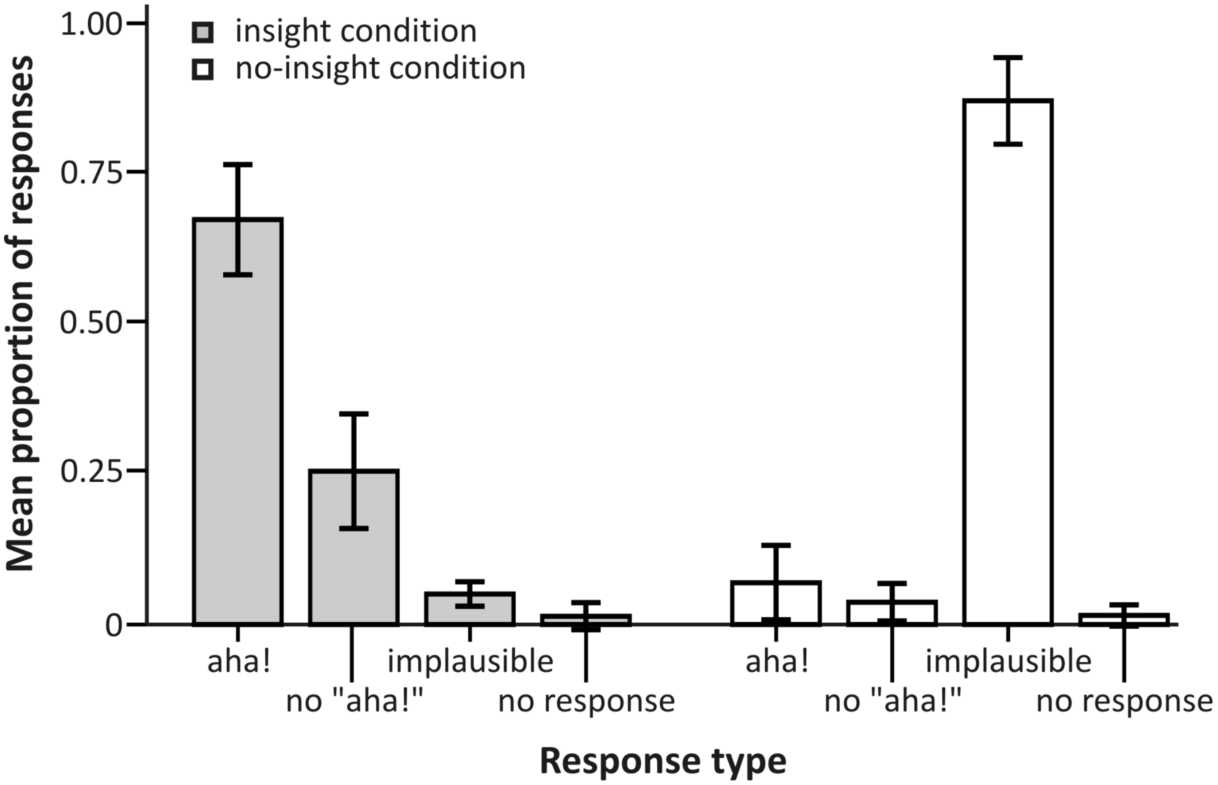

Figure S1. Proportion of responses for each experimental condition. Error bars represent 95 % confidence intervals.

Second, we analyzed memory performance with respect to insight (CRAT) and no-insight (control) condition, without further splitting the conditions depending on ratings of the quality of the responses from the encoding phase (i.e., plausible with “aha!”/ plausible without “aha!”/ implausible). All means and standard deviations are reported in Table S1.

Compared to the no-insight condition, participants correctly recognized more old solutions from the insight condition. A paired *t* test confirmed this difference to be statistically significant [$t(25) = 5.95$, $p < .001$, Cohen's $d = 1.451$]. Moreover, significantly more solutions were remembered from the insight compared to the no-insight condition [$t(25) = 5.47$ $p < .001$, Cohen's $d = 1.862$]. The insight and no-insight conditions did no differ in regard to their rate of "know" responses as supported by a repeated-measures *t* test [$t(25) = 1.20$, $p = .241$, Cohen's $d = 0.303$]. Thus, even in the second round, where insight and no insight conditions were presented one after another, recognition memory only differs for our insight and no-insight conditions due to a higher remember rate for insight, suggesting that insight solutions leave a more detailed memory trace. This finding is all the more important, since the no-insight condition was always presented after the insight condition: One could have expected the difference in remember rates to be much smaller than for the second run, because of retroactive interference.

Table S1. Memory performance (mean proportion of responses) during the test phase 24 hours after the encoding phase for all old items from the second run.

	**Insight (CRAT)**		**No-insight (control)**	
	mean	SD	mean	SD
**Hit**	.48	.15	.32	.18
**remember**	.21	.14	.07	.07
**know**	.28	.09	.25	.15
**Guess**	.03	.08	.05	.07
**Miss**	.48	.17	.63	.21

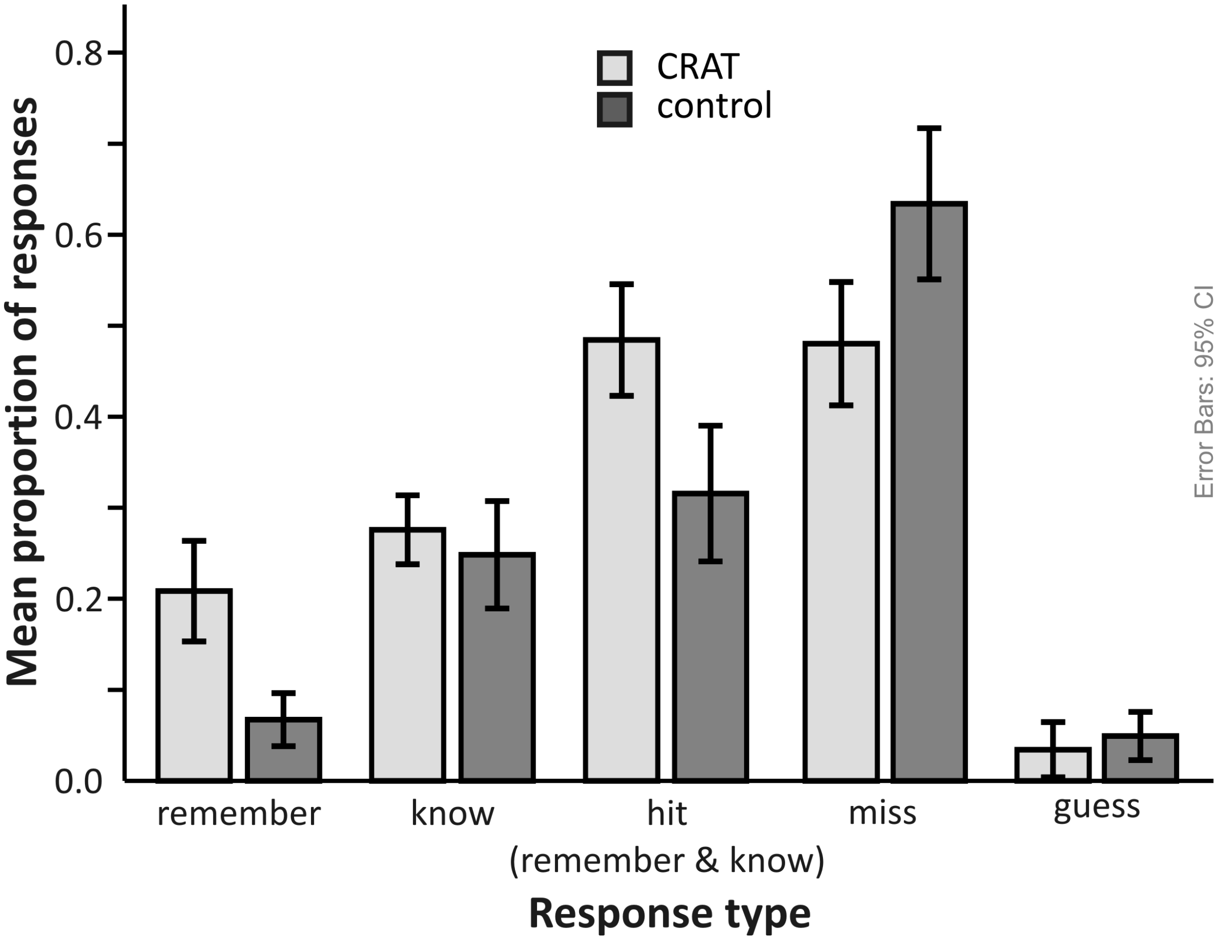

Figure S2. Recognition memory performance (as mean proportion of responses) for Run 2. The result pattern was very similar to Run 1 (cf. Figure 3 of the main text), but the difference between the proportion of remember responses and misses for insight as compared to no-insight items was larger in Run 2.

2.2 fMRI data

For the second run, statistically different activations were found for the processing of triads and the CRA > CTRL contrast (Z-threshold = 2.3, *p* = .05), suggesting that participants noticed at least unconsciously, that they were either in a block of solvable (insight) or unsolvable (no-insight) items. We found activation in visual processing areas, that is, the occipital gyrus, frontal regions, that is, the inferior and medial frontal gyrus, the dorsal striatum (left caudate nucleus, left putamen), and in the right thalamus (Table S2, Figure S3). For the reverse contrast (CTRL > CRA, Figure S4, Table S3), increased activation (Z-threshold = 2.3, *p* = .05) was primarily observed for the fronto-parietal attention network, that is right superior frontal gyrus, the gyrus supramarginalis and the gyrus angularis.

During presentation of solution words, both contrasts (CRA > CTRL, Table S4, Figure S5; CTRL > CRA, Table S5, Figure S6) were overall comparable between both runs. However, in contrast to the first run, no differences were observed for the DM contrasts, that is, the comparison between later recognized and later forgotten items.

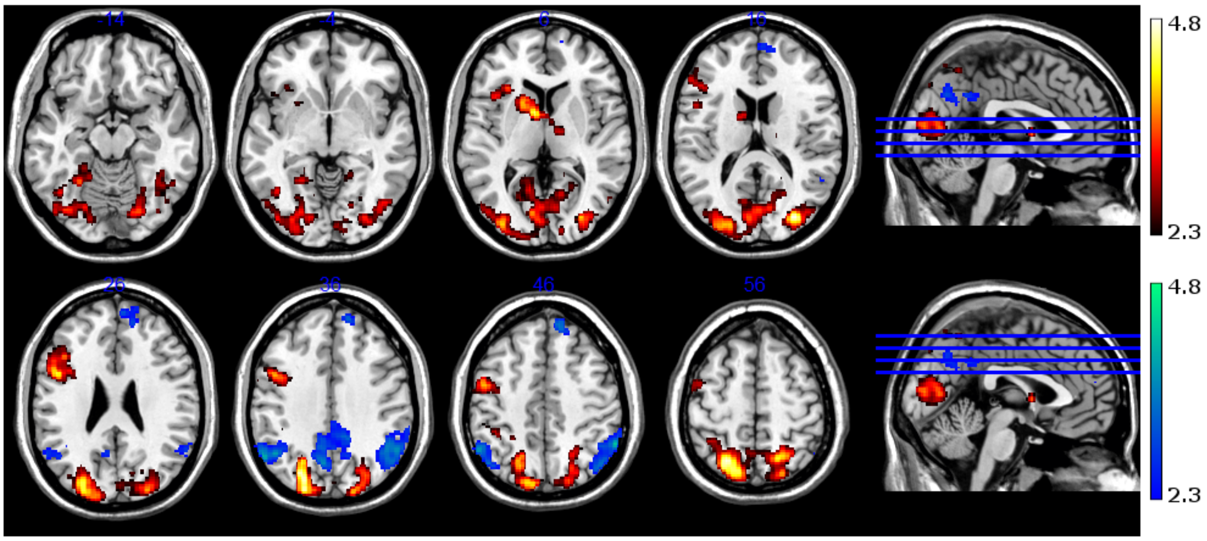

Figure S3. FMRI activation differences between solvable insight and unsolvable no-insight items during the presentation of the problem (second run). Red-yellow activations depict the insight > no-insight contrast and blue activations the reverse contrast.

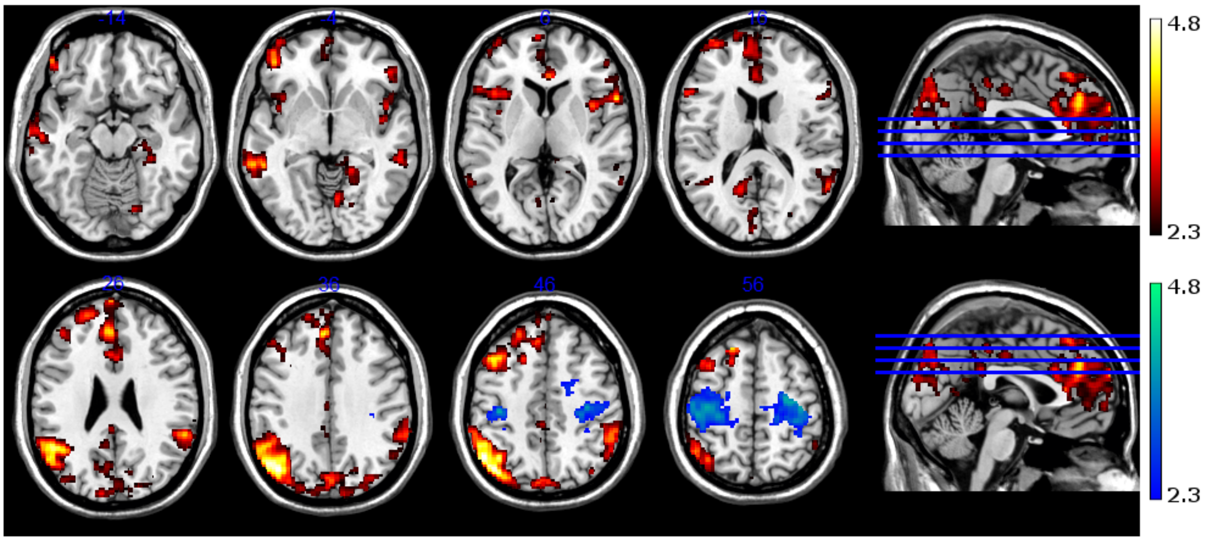

Figure S4. FMRI activation differences between solvable insight and unsolvable no-insight items during the presentation of the solution (second run). Red-yellow activations depict the insight > no-insight contrast and blue activations the reverse contrast.

Table S2. FMRI activation clusters between solvable insight > unsolvable no-insight items during the presentation of the triad (second run, z-threshold = 2.3, p = .05).

						MNI coordinates (mm)		
**cluster & region**		**BA**	**NoV**	***p***	***Z***	**x**	**y**	**z**
1	L lateral occipital cortex, superior region;	5, 7, 17, 18, 19	11531	< .001	29.9	-24 (-4)	-64 (-76)	58 (20)
	L cuneus;							
	L occipital pole;							
	R lateral occipital cortex, superior region							
2	L precentral gyrus;	6, 44, 45, 48	1611	.002	6.62	-46 (-47)	6 (10)	30 (32)
	L inferior frontal gyrus, (opercular part / triangular part);							
	L medial frontal gyrus;							
3	L caudate nucleus;	25, 47, 48	1011	.03	4.34	-8 (-16)	4 (9)	4 (5)
	L putamen;							
	L frontal operculum;							
	L insula;							
	L inferior frontal gyrus, triangular part;							

L temporal pole;							
L orbitofrontal cortex;							
R thalamus							

Table S3. FMRI activation clusters between solvable insight < unsolvable no-insight items during the presentation of the triad (second run, z-threshold = 2.3, p = .05).

					MNI peak coordinates (mm) (center coordinates)		
**cluster & region**	**BA**	**NoV**	***p***	***Z***	**x**	**y**	**z**
1 R angular gyrus; R supramarginal gyrus, posterior region; R lateral occipital cortex, superior region	39, 40	1383	.004	5.79	59 (50)	-50 (-56)	46 (40)
2 L precuneus; L cuneus; L supracalcarine cortex; L cingular gyrus, posterior region	7, 23	868	< .001	3.73	-8 (-2)	-64 (-54)	40 (35)
3 L angular gyrus; L lateral occipital cortex, superior region; L supramarginal gyrus, posterior region	39, 40	856	< .001	3.68	-56 (-49)	-58 (-58)	38 (39)
4 R frontal pole; R superior frontal gyrus	9, 10	547	< .01	2.23	14 (13)	52 (52)	40 (35)

Table S4. FMRI activation clusters between solvable insight > unsolvable no-insight items during the presentation of the solution (second run, z-threshold = 2.3, p = .05).

					MNI peak coordinates (mm)		
**cluster & region**	**BA**	**NoV**	***p***	***Z***	**x**	**y**	**z**
1 L lateral occipital cortex, superior region; L angular gyrus;	7, 18, 19, 22, 39, 40, 41	7313	< .001	5.18	-42 (-34)	-72 (-60)	38 (29)

	L supramarginal gyrus, posterior region							
2	L paracingular gyrus;<br>L superiorer frontal gyrus;<br>L frontal pole;<br>L medial frontal gyrus	9, 10, 24, 32	5575	.008	4.56	-4 (-19)	40 (41)	34 (28)
3	R supramarginal gyrus, posterior region;<br>R angular gyrus;<br>R middle temporal gyrus, temporo-occipital parts;<br>R lateraler occipital cortex, inferior region;<br>R temporal superior gyrus, posterior region	21, 39, 40, 48	1869	.004	4.09	56 (56)	-42 (-46)	42 (29)
4	R inferior frontal gyrus, pars opercularis;<br>R precentral gyrus;<br>R frontal pole;<br>R central opercular cortex;<br>R frontal operculum;<br>R orbitofrontal cortex	45, 48	979	< .001	4.15	58 (51)	14 (20)	6 (4)
5	R cingular gyrus;<br>R thalamus;<br>R temporal fusiform cortex, posterior region;<br>R parahippocampal gyrus, posterior region;<br>R temporal occipital fusiform cortex;<br>Lingual gyrus	27, 37	835	.001	3.58	10 (18)	-36 (-53)	2 (-8)
6	L frontal operculum;<br>L insula;<br>L inferior frontal gyrus, triangular part / opercular part;<br>L medial frontal gyrus;<br>L temporal pole	45, 47, 48	636	.007	4.13	-38 (-44)	18 (17)	2 (3)

Table S5. FMRI activation clusters between solvable insight < unsolvable no-insight items during the presentation of the triad (second run, z-threshold = 2.3, p = .05).

						MNI peak coordinates (mm)		
**cluster & region**		**BA**	**NoV**	***p***	***Z***	**x**	**y**	**z**
1	L postcentral gyrus;<br>L precentral gyrus	2, 3, 4, 6, 40	1285	.02	4.52	-38 (-37)	-24 (-24)	48 (54)
2	R precentral gyrus;<br>R postcentral gyrus;<br>R superior parietal lobe	3, 4, 6	1007	< .001	3.56	38 (34)	-20 (-21)	52 (50)
